# “The missing ingredient”: the patient perspective of health related quality of life in bronchiectasis: a qualitative study

**DOI:** 10.1186/s12890-018-0631-7

**Published:** 2018-05-22

**Authors:** Emily K. Dudgeon, Megan Crichton, James D. Chalmers

**Affiliations:** 1 Scottish Centre for Respiratory Research, University of Dundee, Ninewells Hospital and Medical School, Ninewells Drive, Dundee, DD1 9SY Scotland; 2 Division of Molecular and Clinical Medicine, University of Dundee, Dundee, DD1 9SY UK

**Keywords:** Bronchiectasis, Symptoms, Endpoints, Questionnaires, Qualitative

## Abstract

**Background:**

Bronchiectasis is a heterogeneous disease which affects quality of life. Measuring symptoms and quality of life has proved challenging and research is limited by extrapolation of questionnaires and treatments from other diseases. The objective of this study was to identify the major contributors to quality of life in bronchiectasis and to evaluate existing health related quality of life questionnaires in bronchiectasis.

**Methods:**

Eight adults with bronchiectasis participated in one to one semi-structured interviews. These were recorded and transcribed verbatim. Thematic analysis was used to identify core themes relevant to disease burden and impact. Participant views on current health related quality of life questionnaires were also surveyed.

**Results:**

Bronchiectasis symptoms are highly individual. Core themes identified were symptom burden, symptom variation, personal measurement, quality of life and control of symptoms. Themes contributing to quality of life were: social embarrassment, sleep disturbance, anxiety and modification of daily and future activities. Evaluation of 4 existing questionnaires established their individual strengths and weaknesses. A synthesis of the participants’ perspective identified desirable characteristics to guide future tool development.

**Conclusions:** This qualitative study has identified core themes associated with symptoms and quality of life in bronchiectasis. Current treatments and quality of life tools do not fully address or capture the burden of disease in bronchiectasis from the patients’ perspective.

## Background

Bronchiectasis is a chronic respiratory disease characterised by cough, sputum production and frequent chest infections [1, 2]. These symptoms impact health related quality of life (HRQL). HRQL questionnaires have become a useful tool for measuring the impact of disease on patients’ lives and are essential to assess new treatments in clinical trials [3–5]. HRQL questionnaires have been developed for respiratory conditions such as COPD, asthma and chronic cough [6–9]. There is some overlap between symptoms of bronchiectasis and those of COPD and asthma, and two of these HRQL questionnaires (St George’s Respiratory Questionnaire and Leicester Cough Questionnaire) have been validated for use in bronchiectasis. [3, 7]. However it was not until 2014 that a HRQL questionnaire designed specifically for bronchiectasis was published [10]. There are no large comparative studies to determine which is the best HRQL questionnaire for bronchiectasis.

The quality of life bronchiectasis questionnaire (QoL-B) was developed in the context of a clinical trial of an inhaled antibiotic and has not been tested widely in broad populations of patients with bronchiectasis [11].

In recent years, there has been a shift away from a traditional model of research where doctors or those working in the pharmaceutical industry decide on the best outcome measure when assessing new treatments. The patient led model of research recognises the value in patient involvement at every stage of clinical research, and best practices have now been identified [12]. There have been a series of unsuccessful trials in bronchiectasis. Treatments that are widely used in clinical practice, and believed to be effective by clinicians and patients, may give only small changes in questionnaires, perhaps because we are unable to effectively measure what matters to patients with bronchiectasis [13–16].

A major limitation affecting all bronchiectasis research is that tools, approaches, questionnaires and treatments have generally been extrapolated from other diseases. There have been few studies specifically addressing the opinions, experiences and needs of patients with bronchiectasis.

Bronchiectasis is a distinct, heterogeneous condition in its own right [17]. Quality of life in particular is deeply personal and specific to an individual. Patients’ quality of life may be determined by more than simply the number and frequency of physical symptoms but also by social, psychological and other personal factors [4–9].

In view of the importance of health related quality of life questionnaires for understanding bronchiectasis disease burden and as an outcome in clinical trials, we conducted a qualitative study to determine what contributes to quality of life in bronchiectasis patients and to gather patient views and opinions on how existing questionnaires reflect their quality of life. Finally we present a synthesis of bronchiectasis patients’ evaluation of existing health related quality of life questionnaires, including the identification of desirable characteristics, with the aim of guiding development of more patient focussed, responsive and meaningful HRQL tools in future.

## Methods

We performed a qualitative study of patients with bronchiectasis attending a regional specialist clinic at Ninewells Hospital in Dundee, UK.

The *inclusion criteria* were: A clinical diagnosis of bronchiectasis confirmed by CT scanning, an ability to communicate in English, respiratoy symptoms that are caused by the primary diagnosis of bronchiectasis. Key *exclusion criteria* were: Inability to give informed consent; diagnosis of cystic fibrosis, severe COPD or severe asthma. The study was approved by the North West Ethics Committee- approval number 16-NW-0100. All patients provided written informed consent for participate.

### Study interviews

The study consisted of a single in depth semi-structured interview approximately one hour in length. Interviews explored the nature, variation and impact of symptoms, and the value of existing questionnaires as outlined below. Interviews were audio-recorded and transcribed verbatim. The interviewer was not involved in the clinical care of the participants, and was trained in qualitative methodology but did not have experience in bronchiectasis. This was desirable to avoid conscious or unconscious biases determined by prior experience with bronchiectasis patients.

### Analysis

Transcripts were analysed by the researchers and common themes were identified by thematic analysis. Following Strauss and Corbin (1998) text was analysed line by line [18].

Responses were initially coded and grouped according to the research objectives [19]. Common themes and responses were identified. The researchers modified their coding and groups according to participant responses. Interviews were participant driven, with the researcher attending to understanding participants’ perspectives from their point of view and using terminology common to participants identified through the interviews. Sample size was determined empirically, and was terminated at participant 8 after reaching data saturation, in which no new themes were identified.

The primary outcome of the study was to understand the symptom burden of bronchiectasis and the key determinants of quality of life. Secondary objectives were to evaluate those symptoms that change most frequently with exacerbations. Finally the study aimed to evaluate how well existing questionnaires captured participants’ symptoms and quality of life, and the accessibility and ease of use of questionnaires from a patient perspective. The interview schedule which addresses each of these objectives is shown in Table 1.

**Table 1:** Interview outline

Symptom burden What daily symptoms of bronchiectasis do you experience? How do symptoms vary from day to day? Is there a way to quantify changes- how do participants express differences in how you are feeling (to doctors and to other patients)? Exacerbations What changes when you have an exacerbation? What are the key symptoms that lead you to seek medical help? Existing questionnaires How well do these reflect your symptoms and the changes in your symptoms? Do you find the questionnaires easy to understand and answer? How could you improve them?	

## Questionnaires

Participants were presented with the questionnaires at least 24 h before the interview in order to have time to become familiar with and to complete the questionnaires.

The questionnaires selected for use in this study were based on those identified in a systematic review of the literature as being used in bronchiectasis studies to evaluate symptoms or quality of life. These were

- *St. Georges Respiratory Questionnaire* [3]
- *Quality of life bronchiectasis questionnaire version 3.1* [4]
- *Leicester Cough Questionnaire* [7]
- *COPD assessment test* [20]

The St George’s Respiratory Questionnaire is a 50 item tool with 2–5 responses per item (mean 2.5), 5 A4 pages in length, giving a score of 0–100 points where 0 is no impairment of quality of life and 100 is maximum impairment. We note that there are 3 versions each with a different recall time (1 month, 3 months and 1 year), and the 3 month version was used in this study.

Quality of Life Bronchiectasis questionnaire is a 37 item tool with 2–6 responses per item (mean 4.1), 3 A4 pages in length, giving a score from 0 to 100 in each of 8 domains (respiratory symptoms, physical, role, emotional and social, vitality, health perceptions, treatment burden) and overall, where 0 is maximum impairment of quality of life and 100 is no impairment. It has a recall time of 1 week.

Leicester Cough Questionnaire is a 19 item tool with 7 responses per item on one A4 page, giving a score of 1–7 for each of 3 domains- physical, psychological and social and a total score of 3–21 with a higher score indicating minimal impairment on quality of life. It has a 24 h recall time.

COPD Assessment Test is an 8 item tool with 6 numerical responses per item, on one A4 page, giving a score out of 40. A higher score suggests a greater impact on quality of life. It has no specified recall time.

People living with bronchiectasis are referred to as patients and those who were interviewed for this study will be referred to as participants.

## Results

Eleven consecutive patients were invited to participate and 8 interviews were carried out (5 female, 3 male). The mean age was 72 (63–80). 4 had idiopathic bronchiectasis, 2 had post-infective bronchiectasis. 1 participant had co-existing COPD and 1 participant had co-existing mild asthma (Table 2).

**Table 2:** Participant characteristics

Characteristics	N (%) or median (IQR)
N	8
Age- mean-range	72 (range 63–80)
Gender	5/8 (62.5%) female
Smoking history	6/8 (75%) never smokers
FEV1% predicted (mean-sd)	71.6% (24.4)
Bronchiectasis severity index (mean-sd)	8.6 (4.4)
Cause of bronchiectasis	
Idiopathic	4 (50%)
Post-infective	2 (25%)
Sjogrens syndrome	1 (12.5%)
Ulcerative colitis	1 (12.5%)
Exacerbations per year (mean-sd)	1.8 (1.3)
*Pseudomonas aeruginosa* infection	3 (38%)
Long term macrolide use	4 (50%)

**Fig. 1:**
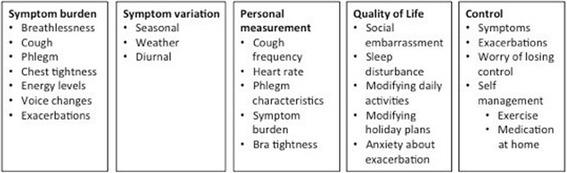
Core themes and sub themes identified from interviews

Thematic analysis of the interviews identified 5 key determinants of symptom burden and quality of life. Although our pre-specified analysis had intended to consider exacerbation impact separately from stable disease burden, our interviews revealed that participants regarded exacerbations as an integral part of daily disease impact. Participants did not regard exacerbations as a separate state from stable disease, but rather a continuum where daily symptoms become more severe or persistent. Participants defined an exacerbation as a worsening of symptoms, and recognised that this means they need to seek medical help, however, sometimes patients do not seek medical help and try to self-manage. In addition, all participants reported that exacerbations impacted on daily quality of life even when “well” because of anxiety around exacerbations and the modifying of activity and future plans due to risk of exacerbations (Fig. 1).

Therefore exacerbations have been included in the following analysis as part of symptom burden. Table 3 shows an example of the analysis whereby individual responses were coded and then grouped into common themes.

**Table 3:** Example of the coding and grouping approaches for analysis

Participant information	Coding	Common theme
“I have like a film forming across my chest”	Chest tightness	Symptom burden
“Coughing usually starts about twelve O’Clock and it doesn’t have any rhyme or reason”	Diurnal variation	Symptom variation
“Right, if somebody comes round to your house, you get a visitor who goes ‘I’m not feeling well’ then I just say ‘well go away, just go, stay away from me’.”	Social anxiety	Quality of life
“how do you define moderate difficulty and a little difficulty”	Questionnaire answers	Questionnaires

The themes included were symptom burden, symptom variation, personal measurement of symptoms, quality of life and control. Symptom burden, symptom variation, and quality of life were pre-specified terms while personal measurement of symptoms and control were added based on consistent reporting by participants.

### Theme 1: Symptom burden

A combination of cough, breathlessness and sputum production was present in all participants although the relative importance of each of these symptoms was highly variable when describing the impact on their quality of life.

Participant 4 **“**So, yeah, that, bronchiectasis, its, the biggest thing is breathlessness.”

Participant 5 “The main one is that I, I cough a lot, and I cough a lot of phlegm up, erm I’m also very, I feel very short of breath sometimes.”

5/8 described chest tightness as a prominent symptom in addition to breathlessness, cough and sputum production.

Participant 7 “I have like a film forms across my chest.”

Additional symptoms that were reported were decreased energy levels (7/8), swallowing difficulties (3/8) and hoarse voice (2/8).

Exacerbations were commonly (7/8) described as an increase in symptom burden accompanied by a feeling of being generally unwell.

Participant 1 “Erm, just general feeling not good, you know, and tired, and erm breathless, erm a lot more phlegm, using my inhaler a lot more”

Another participant described their exacerbations much more in terms of change in character of cough and increased sputum purulence, without necessarily feeling generally unwell.

Increased sputum purulence is regarded by guidelines as a core symptom of exacerbation. In this cohort, change in sputum colour was mentioned as a key symptom of exacerbation in only 4/8 participants. Participants described changes in sputum in many different ways using taste, volume, viscosity and colour with each giving different weight to each character.

Participant 7 “The mucus gets really tacky and it doesnae (does not) clear”

### Theme 2: Symptom variation

Participants (5/8) commonly experienced diurnal variation in their symptoms. For some participants symptoms were worse in the morning, while for others they were worse in the afternoon or evening.

Participant 6 “I don’t seem to have a problem until about 4 o clock in the afternoon… Yeah I do tend to avoid, meeting people you know, between four [pm] and six [pm].”

Environmental factors such as the weather, smoke, dust and paint also affected participants’ symptoms.

Participant 7 “I like, like going to watch the football, but if it, if it’s a damp rainy cold night then I’m no going. I’ll just say nah because I’ll feel really horrendous the next day.”

There was no characteristic pattern to participants’ symptoms with the diurnal variation being highly individual.

### Theme 3: Personal measurement of symptoms

During the interviews it became clear that participants monitor their symptoms in different ways. Participants often expressed this in terms of the difference between a good day and a bad day. Most participants (6/8) had their own individual way of measuring how they are on any given day.

Interviewer: is there anything else that you can measure how bad you’re feeling on one specific day? Participant 1: “It’s a strange one. My bra gets tight. [laughs]”.

Participant 3 “I know I’m getting an infection if it [phlegm] goes through a colour change and my pulse rate goes up. My pulse rate is normally about 58/60 and that goes 70/75.”

Some participants (4/8) know when an exacerbation is coming on because of symptoms that consistently occur at the onset.

Participant 2 “When I have an exacerbation, yes, I tend first of all to start getting hot and cold flushes, …I then start to become dizzy. I start to cough a lot more.”

The other participants had more heterogeneous, unpredictable events without characteristic symptoms at onset.

### Theme 4: Quality of life

All of the participants agreed that the disease had a major impact on their quality of life. Impacts on quality life were diverse, taking in social embarrassment associated with cough and sputum, sleep disturbance, modification of activities and holiday plans, and anxiety or concern about developing exacerbations.

Participants feel embarrassed about sputum production in public.

Participant 5 “I’m worried about that [coughing when talking to someone] because 1) I don’t like to do it 2) they might think its unhygienic and erm 3) I do think its unhygienic myself.”

Participants feel they have to explain their symptoms.

Participant 4 “I have come out of church a couple of times and it upsets people because they think is she going to die out there or whatever.”

Participant 5 “whoever it is will think you’re giving them the bug of death or something you know.”

Symptoms also cause participants to avoid certain situations.

Participant 7 “I wouldnae (would not) want to go to the pictures or a theatre… It would spoil other people’s enjoyment.”

Symptoms during the night can cause significant sleep disturbance, with several participants sleeping in separate rooms to their partner so as not to disturb them.

Participant 2 “I do cough a lot…especially at night time trying to get to sleep. That, erm, is a concern for me, not to unduly disturb my wife.”

Symptom burden and seasonal and diurnal symptom variation has forced many participants to modify their daily activities.

Participant 7 “as I say you can’t go in the winter months you cannae go out the walking that you do so you’re confined to the house a wee bitty more, so you get a wee bitty fed up so you munch a wee bitty more and you put on a bit more weight that you’ve just took off.”

The unpredictability of an exacerbation causes significant anxiety for participants and their families, particularly around planning travel and family events. For example, the word anxiety was mentioned 21 times by a single participant.

### Theme 5: Control

Lack of control over symptoms was consistently reported (5/8) as a key impact of the disease. Control was frequently (7/8) mentioned in interviews and only one participant felt they were always in control of their condition. One participant cited control as the one thing they would change about the condition if they could.

Participant 4 “ I don’t have control over my cough…I mean you can grab the bottle of water and hope it shuts up for a minute or two but it’s not, you know, I don’t feel I control it all.”

Regular exercise (4/8) and having antibiotics at home to self-manage exacerbations made participants feel that they had more control over their condition.

Participant 1 (regarding self-management with antibiotics at home) “And you feel as if you’ve got control. You know, that you can do something. Cos if the doctor’s surgery is closed over the weekend, what do you do?”

Exacerbations can take away the feeling of having control which can cause anxiety.

Participant 2 “Well I feel very dependent on others. And that to some extent is debilitating. It’s almost humiliating at times.”

### Evaluation of questionnaires

**Fig. 2:**
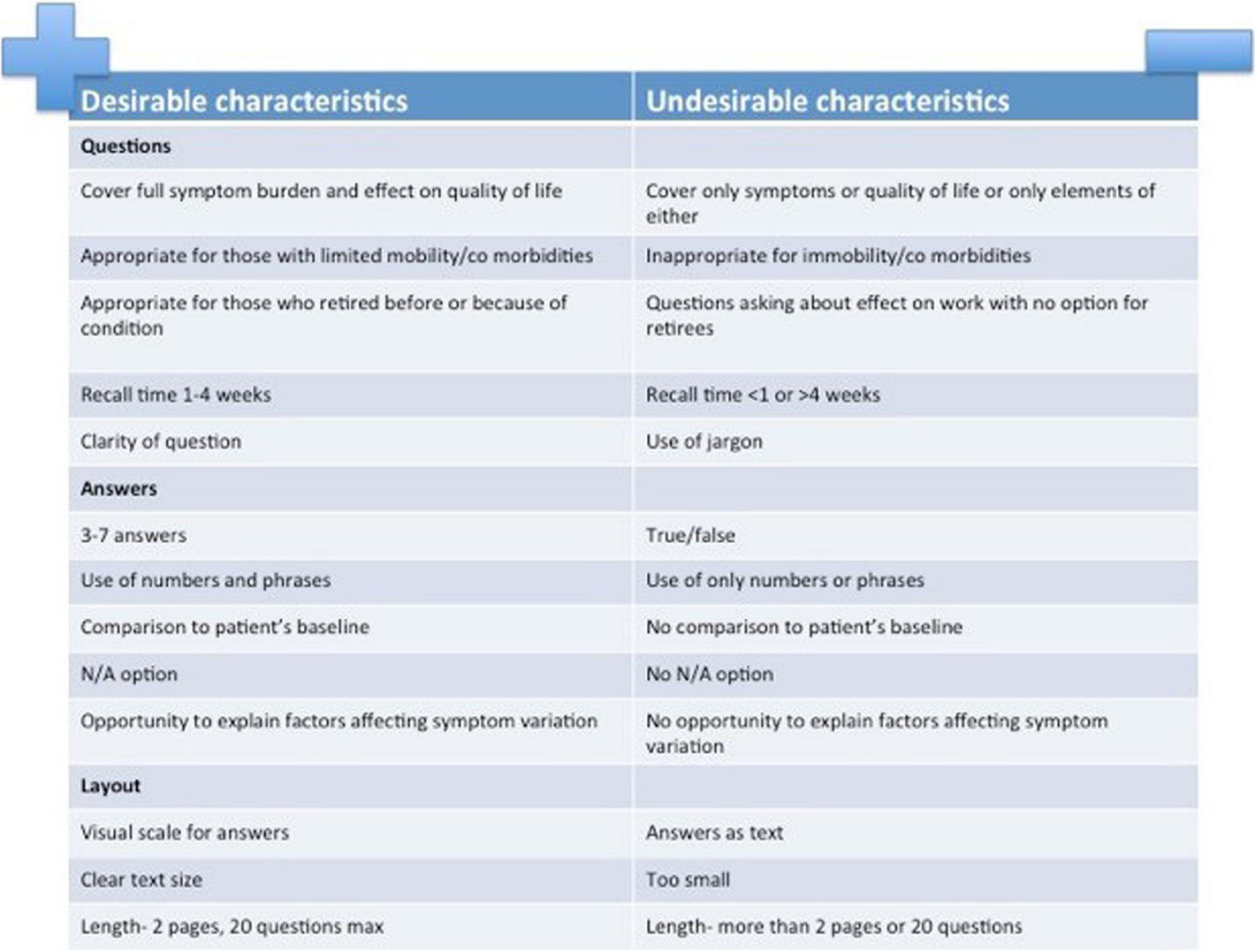
Participants’ perspective on different health related quality of life and symptom questionnaires in bronchiectasis

Evaluation of existing questionnaires identified desirable and undesirable characteristics (Fig. 2) for HRQL questionnaires used in bronchiectasis. Participants commented on the extent to which questions were understandable and reflective of their experience, the extent to which answer options gave them scope to express how they felt and the layout of questions in terms of ease of use and time taken for completion. Participants varied in their knowledge of medical terms. For example, commonly used terms like wheeze were considered jargon and poorly understood by many participants.

Figure 2 shows the aspects of questionnaires that participants did and did not value.

#### Referring to specific questionnaires:

SGRQ- Participants reported that true and false answering method was very clear but gave too limited scope for answering questions, and suggested the use of a baseline. The questionnaire requires a recall time of 3 months which concerned some participants.

Participant 5 “again, the true and false, is just, it’s not, you’re not giving enough information to people.”.

Participant 4 “Over the past 3 months, in an average week how many good days? Its, it’s a long time to remember”

QolB- This was the most commonly preferred questionnaire (6/8 participants). The number of multiple choice answers were viewed favourably when compared to the true and false of the SGRQ and the seven choices of the LCQ, but participants felt the questions were sometimes ambiguous. While some participants felt that seven choices were too many, others viewed the increased number of options as favourable.

Participant 7 “During the past week indicate how often you have felt well. Again relative to what? What’s your baseline? The word “well”, is meaningless. It is its meaningless. No I’m no as well as I should be but am I as bad as I could be? No so what’s well?”

CAT- The layout was praised for its simplicity and ease of reading but there was disagreement as to whether the visual scale from 0 to 5 was easy or difficult to answer.

Participant 2 “I like the layout… It’s very visual.”

Participant 4 “I found it very difficult to judge erm, which one, sort of, represented it”

LCQ- The layout was criticised but compared with the other questionnaires, the LCQ’s answers have numbers and phrases which was considered favourable.

Participant 6 “Well it gave you more choices, there was, there was seven choices but it gave you much more, you could more accurately describe what your symptoms were.”

**Fig. 3:**
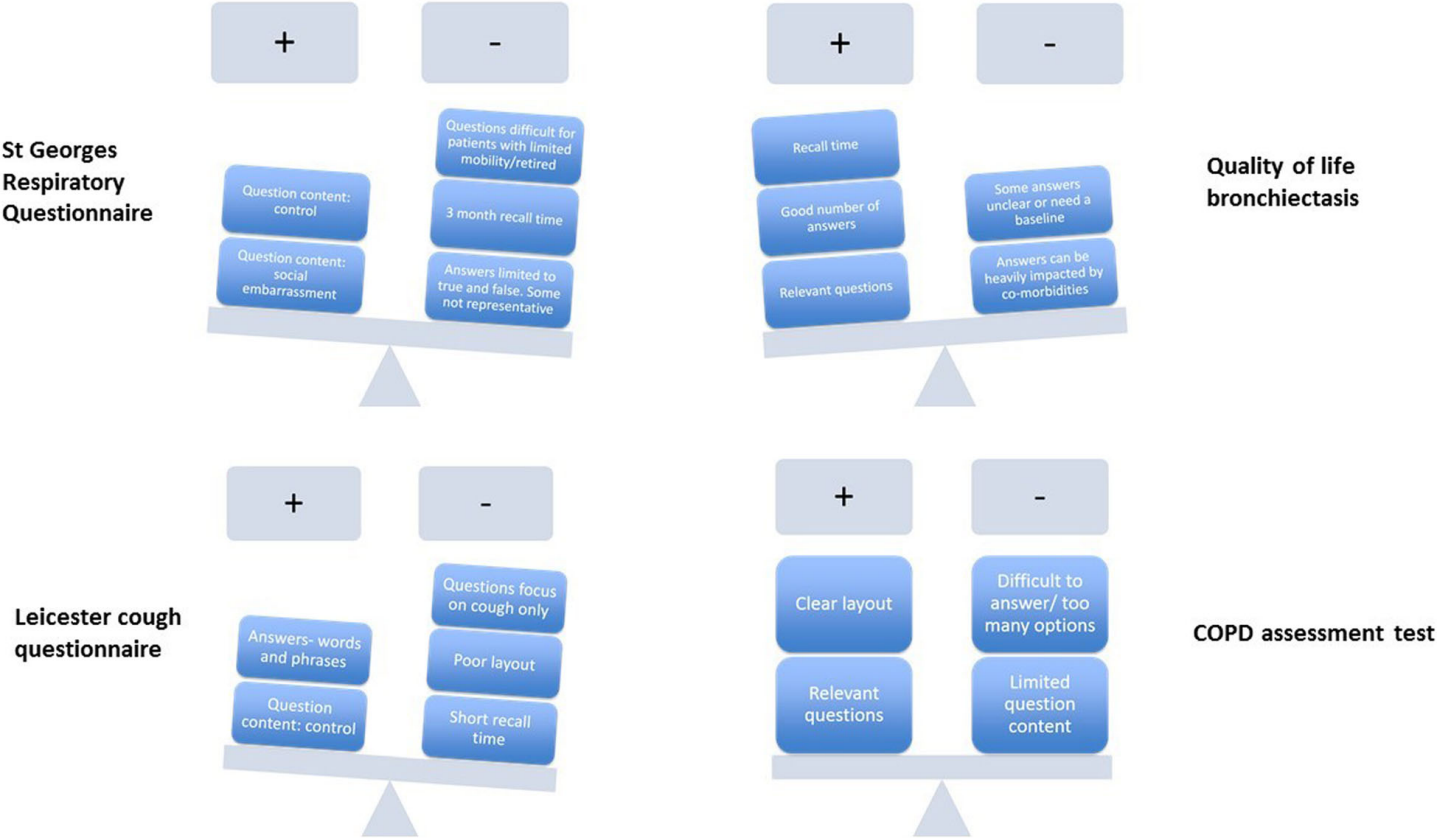
Graphical illustration of the strengths and weaknesses of the different QOL questionnaires based on participants’ evaluation

Overall, the strengths and weaknesses of the different questionnaires from the bronchiectasis patient’s perspective is summarised in Fig. 3.

## Discussion

This qualitative study of symptom burden and quality of life in bronchiectasis has identified a disconnect between the classic symptoms of bronchiectasis (such as sputum production, purulence and exacerbations) and the impact on patients’ quality of life. Our analysis suggests that what most strongly affects a patient’s quality of life is highly personal to the individual, but includes an ability to feel in control of their symptoms, to achieve normal sleep and take part in social activities without embarrassment. Anxiety and fear of exacerbations had a major impact on quality of life.

These findings are important for clinical care, because many of these are aspects that are not frequently explored in a doctor-patient consultation. They are important for the development of new therapies because treatments aiming to improve quality of life need to be capable of addressing the major determinants of quality of life [21, 22].

Quality of life tools are used in clinical practice and in clinical trials to measure disease impact and response to therapy. We conducted what to the best of our knowledge is the only comparative “preference” study relating to quality of life tools in bronchiectasis. This analysis found that each of the questionnaires have different strengths and weaknesses. Discussion of these has allowed us to develop a framework for the “perfect” quality of life tool from a bronchiectasis patient’s perspective. We identified that the quality of life bronchiectasis questionnaire was the most frequently preferred questionnaire from a patient perspective. It should be noted that the clinical value of a questionnaire includes its repeatability, responsiveness and clinical utility and that patient preference and ease of use is only one aspect of the evaluating a questionnaire [3, 4].

An interesting finding was disparity between how patients describe symptoms and how they are evaluated in questionnaires. A question may try to quantify exercise limitation in terms of mild or moderate difficulty, whereas patients do not think about symptoms in this way. Patients were consistently more focussed on “change from baseline” or differences between what they can achieve and what they want to achieve, which is highly individual. It is intuitively correct, and was expressed by the majority of patients, that you cannot accurately quantify something without a frame of reference. Patients find it much more straightforward to say they are “worse than usual” than to say they have “moderate difficulty” carrying out a task, without a frame of reference for how much difficulty a person without bronchiectasis might experience.

It is not surprising that bronchiectasis symptoms and quality of life determinants are heterogeneous because the disease itself is heterogeneous. It is caused by a range of underlying disorders, affecting all age groups and having a highly variable clinical course [23–26]. This emphasises one of the key findings of this research- it may be impossible to fully capture disease impact with categorical scales that do not account for patient’s highly variable baseline symptoms, expectations and co-morbidities [22, 26]. As mentioned above, patients reported that anchoring questions within patients own baseline function could provide a solution to this heterogeneity. An example of an anchored question would be:

How is your breathlessness at the moment?

- My breathless is much better than normal
- My breathless is better than normal
- My breathless is normal for me
- My breathlessness is worse than normal for me
- My breathlessness is much worse than normal

compared to an unanchored question such as:

Walking up a flight of stairs makes me feel breathless.

- True
- False

Our study suggested the “perfect” questionnaire would use both anchored and unanchored questions to establish the patients baseline with a second question or set of questions to establish change from baseline.

There are similarities between our findings and those of qualitative studies in COPD and asthma in terms of symptom burden, anxiety, the benefit of exercise, control and self monitoring [27, 28]. It is interesting to note that the worry of asthma attacks is similar to that of exacerbations in bronchiectasis in impacting quality of life even when patients are not experiencing symptoms. Although it was not identified as a major theme, control was discussed in both the COPD and asthma studies. Similar to the current study, it was mentioned in a number of contexts: for example in asthma patients not being able to control the external environment leading to exposure to triggers and in COPD patients trying to take control of their condition.

Self monitoring differed between asthma and bronchiectasis patients. Whereas asthma patients are able to use the objective measure of peak expiratory flow rate, bronchiectasis patients have no objective measurement of their symptoms. As a result, self monitoring tends to be more subjective, and more individualised in bronchiectasis.

The COPD study reported that objective measurement of severity does not correlate with patient experience. The authors hypothesise that this may be attributable to variations in coping strategies and self management, and that patients with poor quality of life scores may be most suitable for non pharmacological interventions. The use of data measuring patient reported impact on quality of life in guiding management is an interesting suggestion, particularly as medicine and clinical research transition from a traditional paternalistic style to a patient led model.

Another qualitative study compared 3 quality of life questionnaires used in asthma [29]. Participants identified missing and irrelevant content when assessing questionnaires as weaknesses. Similar to the current study, confusing questions were identified as a weakness in several questionnaires and the questionnaire preferred by participants was one that covered both medical and psychosocial impact of disease. This is in line with our findings on how bronchiectasis impacts quality of life.

Therefore our findings are consistent with work in other chronic respiratory conditions but with disease specific features because of the subtle differences in the combination of symptoms present in each disease.

Limitations of this study must be acknowledged. This is a qualitative study and as is typical of such studies the sample size is small. This study is single centre and it is known that bronchiectasis can be quite heterogeneous across different healthcare systems. Nevertheless our patient population is typical/representative in terms of demographics of European bronchiectasis cohorts. [26, 30, 31] A small number of patients had previously completed questionnaires such as the QOL-B as part of clinical research studies and so we acknowledge prior experience as a potential source of bias. The length of interview and timing of interviews during working hours may have skewed the population towards older, retired participants. Nevertheless, as the average age of bronchiectasis patients is 65–70 years, we do not regard this as major bias [31, 32].

## Conclusions

This study has characterised bronchiectasis symptom burden and its impact on quality of life and identified scope for improving existing health related quality of life questionnaires. [32] The framework we have developed can be used to evaluate future HRQL questionnaires for bronchiectasis.

## Abbreviations

CAT: COPD assessment test
COPD: Chronic obstructive pulmonary disease
CT: Computed tomography
FEV1: Forced expiratory volume in 1 s
HRQL: Health related quality of life
LCQ: Leicester cough questionnaire
Qol-B: Quality of life bronchiectasis
SGRQ: St Georges Respiratory Questionnaire
